# Molecular recognition of flunarizine dihydrochloride and β-cyclodextrin inclusion complex by NMR and computational approaches

**DOI:** 10.1186/s13065-018-0395-4

**Published:** 2018-03-28

**Authors:** Santosh Kumar Upadhyay, Syed Mashhood Ali

**Affiliations:** 1 CSIR-Institute of Genomics & Integrative Biology, New Delhi, 110020 India; 2 Department of Chemistry, Aligarh Muslim University, Aligarh, 202002 UP India

**Keywords:** Flunarizine dihydrochloride, β-Cyclodextrin, Inclusion complex, NMR spectroscopy, Molecular recognition, ROESY, Molecular docking

## Abstract

**Background:**

Flunarizine dihydrochloride (FLN) is used in the prophylactic treatment of migraine, vertigo, occlusive peripheral vascular disease and epilepsy. Cyclodextrins (CDs) are chiral, truncated cone shaped macrocycles known for their inner hydrophobic and outer hydrophilic site. They form complexes with hydrophobic drug molecules and enhance the solubility and bioavailability of such compounds by enhancing drug permeability through mucosal tissues. NMR spectroscopy and computational docking have been recognized as an important tool for the interaction study of CDs-drug inclusion complexes in solution state.

**Results:**

The structural assignments of FLN and β-CD protons were determined by ^1^H NMR and 2D ^1^H-^1^H COSY NMR spectroscopy. ^1^H NMR spectroscopic studies of FLN, β-CD and their mixtures confirmed the formation of β-CD-FLN inclusion complex in solution. ^1^H NMR titration data for β-CD-FLN inclusion complex showed 1:1 stoichiometry, an association constant of *K*_*a*_ = 157 M^−1^ and change in Gibbs free energy of ∆G = − 12.65 kJ mol^−1^. The binding constant of the β-CD inclusion complex with two nearly similar structures, FLN and cetirizine dihydrochloride, were compared. Two-dimensional ^1^H-^1^H ROESY spectral data and molecular docking studies showed the modes of penetration of the aromatic rings from the wider rim side into the β-CD cavity. The possible geometrical structures of the β-CD-FLN inclusion complex have been proposed in which aromatic rings protrude close to the narrower rim of the β-CD truncated cone.

**Conclusion:**

NMR spectroscopic studies of FLN, β-CD and FLN:β-CD mixtures confirmed the formation of 1:1 inclusion complex in solution at room temperature. Two-dimensional ^1^H-^1^H ROESY together with molecular docking study confirmed that the F-substituted aromatic ring of FLN penetrates into β-CD truncated cone and the tail of aromatic rings were proximal to narrower rim of β-CD. The splitting of aromatic signals of FLN in the presence of β-CD suggests chiral differentiation of the guest FLN by β-CD.
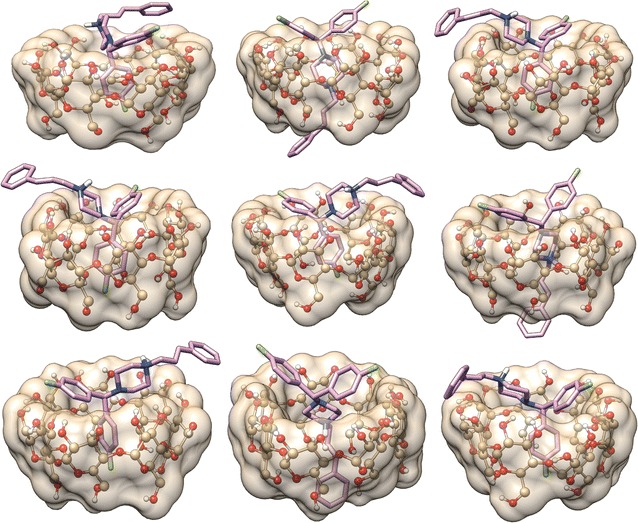

**Electronic supplementary material:**

The online version of this article (10.1186/s13065-018-0395-4) contains supplementary material, which is available to authorized users.

## Introduction

**Fig. 1:**
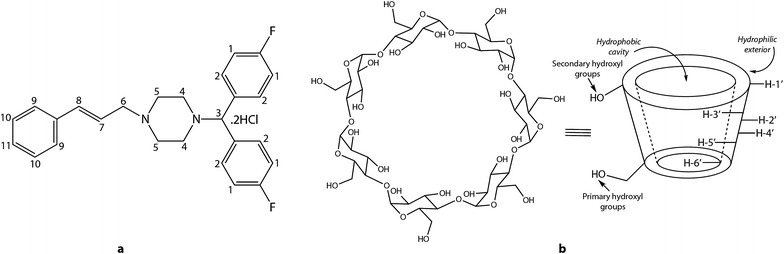
Structural representation of **a** FLN (guest) ligand and **b** β-CD (host) macrocycle [*Source* Adopted from “NMR and molecular modelling studies on the interaction of fluconazole with β-cyclodextrin” by S.K. Upadhyay et al. (2009) Chemistry Central Journal 3:9]

Migraine is a severe headache often unilateral, commonly accompanied by nausea, vomiting, and extreme sensitivity to sound and light. Flunarizine dihydrochloride (FLN) is a large hydrophobic fluorinated piperazine derivative, used in the prophylactic treatment of migraine, vertigo, occlusive peripheral vascular disease and epilepsy [1]. FLN (Fig. 1a) is a di-fluorinated derivative of cinnarizine and a poorly water-soluble drug. FLN is a selective calcium entry blocker with calmodulin binding properties and histamine H_1_ blocking activity. It is also known to prevent hepatitis C virus membrane fusion in a genotype-dependent manner [2] and to suppress endothelial angiopoietin-2 in a calcium-dependent fashion in sepsis [3]. FLN is reportedly effective against hepatitis C virus activity, preferably for the genotype 2 viruses [4].

Cyclodextrins (CDs) or cycloamyloses are truncated cone-shaped macrocycles (Fig. 1b) produced from starch through enzymatic degradation. CDs are a family of cyclic oligosaccharides and have been studied extensively as supramolecular hosts [5, 6]. The three common CDs are crystalline, homogeneous, nonhygroscopic substances, consisting of six (α-), seven (β-), and eight (γ-) d-glucose units, respectively, linked by α-d-(1→4) glycosidic bonds (Fig. 1b) [5, 6]. The glucose residue in CD has 4C_1_ (chair) conformation [5]. The primary hydroxyl groups (n) are located at the narrower rim whereas the wider rim is lined with secondary hydroxyl groups (2n). The outer surfaces of the CDs are highly hydrophilic due to the presence of a large number of hydroxyl groups but the central cavities are relatively hydrophobic (Fig. 1b). The outer dimension of these three common CDs are constant at 0.78 nm but their inner dimensions are variable, being 0.57 nm for α-, 0.78 nm for β-, and 0.95 nm for γ-CD respectively [6]. The H-3′ and H-5′ protons of these CDs are located in the hydrophobic central cavity whereas other protons (H-1′, H-2′, H-4′ and H-6′) are located at the outer surface (Fig. 1b), which is relatively hydrophilic. These properties facilitate their aqueous solubility and ability to encapsulate hydrophobic moieties within their central cavities through non-covalent interactions. CDs form host–guest inclusion complexes upon penetration of guest molecule in the central cavity of host CDs.

CDs are extensively studied in various areas of chemistry including macrocyclic [7], supramolecular [8, 9], agro [10], click [11], analytical [12], chromatography [13, 14], sugar-based surfactants [15], foods [16], catalysis [17, 18], membranes [19], textiles [20], cosmetics [21, 22], fragrance and aromas [23, 24], enzyme technology [25], pharmacy and medicine [26–28], microencapsulation [29], nanotechnologies [30–33], remediation [34], decontamination [35] and biotechnology [36]. The unique properties of CDs allow their various applications in many areas [37–40]. CDs are used to prepare inclusion complexes with pharmaceuticals for biomedical applications and biomedicine [22, 31, 36–38]. CDs are widely used in food industry as food additives, stabilizing flavours, to remove undesirable compounds such as cholesterol, and also as agents to avoid microbiological contaminations in the food [16]. CDs can be used to enhance solubility, bioavailability and stability of pharmaceuticals [41–43]. Upon complexation with pharmaceutical compounds, CDs form inclusion complexes with the ability to alter the physiochemical properties of the complexed drug. Various drugs such as nimesulide, omeprazole, piroxicam, mitomycin, diclofenac sodium, indomethacin and others complexed with CDs are approved and available in the market [42]. Inclusion complexes with dimethyl-β-CD are used in the preparation of vaccine Deptacel (Sanofi Group, Pasteur) for protection against diphtheria, tetanus and pertussis. CDs are also used to stabilize sensitive substances to light or oxygen [44], proteins [45], nanoparticles [46], and add value addition of taste and colour of toothpaste [44].

Among various known spectroscopic methods such as Ultraviolet–visible (UV–Vis), Fourier-transform infrared (FTIR) spectroscopy for the studies of inclusion complexes between host CDs and guest molecules, Nuclear Magnetic Resonance (NMR) spectroscopy is considered as one of the most significant analytical tool for understanding the interaction between host and guest molecules [47]. This technique provides not only the structural assignments of host and guest molecules but also data on the inclusion complex formation. Further NMR spectroscopy could also offer valuable information on chiral recognition or chiral discrimination or both [47–49]. NMR titration data can be used to determine the stoichiometry and association constant of the host–guest complexes [50–52].

Two-dimensional (2D) NMR method such as ^1^H-^1^H COSY (COrrelation SpectroscopY) is a useful technique, which provides information on the ^1^H signals arising from neighbouring protons connected through bonds and protons signals emerging from up to 4 bonds can be captured. Two-dimensional ^1^H-^1^H Rotating-frame Overhauser Effect SpectroscopY (ROESY) has been found to be useful for the investigation of the interaction between CD and guest molecule as the Nuclear Overhausser Effect (NOE) cross-peaks are observed between the protons that are close in space even if they are not bonded [47, 50–52]. Two-dimensional ^1^H-^1^H ROESY provides useful information about the location and depth of inclusion of guest molecule into CD cavity [47, 50–52].

The formation of inclusion complex of a guest molecule with CDs results in the ^1^H chemical shift changes (∆δ) in both the host and guest protons. The inclusion of a molecule inside the hydrophobic cavity of CD is mainly characterized by the chemical shift variation of the CD protons located inside the central cavity (H-3′ and H-5′), whereas other CD protons (H-1′, H-2′, H-4′ and H-6′) are less affected. During host–guest inclusion complex formation the guest molecule protons generally show downfield chemical shift changes but sometimes upfield chemical shift changes are also observed [47].

These analytical procedures revealing the structural details of complexes are used in pharmaceutical industries for characterization. In order to understand correct inclusion architecture of interaction between guest FLN and host β-CD, we report here a high-resolution NMR spectroscopic and computer-based molecular docking study. We describe our results based on the ^1^H NMR spectral data with chemical shift changes, 2D ^1^H-^1^H COSY spectrum for assignment of protons and ^1^H-^1^H ROESY spectrum together with molecular docking approaches thus elucidating the structure of the β-CD-FLN inclusion complex.

## Materials and methods

### Materials

Flunarizine dihydrochloride (FLN) was a kind gift from Geno Pharmaceutical Ltd. India. β-cyclodextrin (β-CD) was obtained from Geertrui Haest, Cerestar Application Centre, Food & Pharma Specialities, France. These materials were used as obtained.

### NMR spectroscopy

^1^H NMR and 2D ^1^H-^1^H NMR (COSY, ROESY) spectra were recorded on a JEOL α-500 MHz instrument in D_2_O. The sample temperature was maintained at 300 K during all NMR experiments. The mixing time (*τ*_mix_) for 2D ^1^H-^1^H ROESY spectra was 500 ms under the spinlock condition using standard ^1^H-^1^H ROESY pulse sequences. The chemical shift values (δ) are reported in ppm. No external indicator was used and HDO peak at 4.80 ppm was considered as an internal reference throughout this work. ^1^H NMR spectra of six samples of mixtures of β-CD and FLN with FLN/β-CD molar ratios ranging from 0.2 to 1.8 were recorded. The FLN/β-CD molar ratios were calculated by direct NMR integration of their appropriate signals. The concentration of β-CD was kept constant at 10 mM while that of FLN was varied from 2.0 to 18.0 mM. Chemical shifts changes (Δδ) were calculated according to the formula: $\Delta\delta \, = \,\delta_{{({\text{complex}})}} {-} \,\delta_{{({\text{free}})}}$

### Molecular docking studies

Molecular docking studies were performed using Autodock Vina 1.1.2 [53]. Three-dimensional coordinates of β-CD (PDB Id: 1DMB) were sourced from http://www.rcsb.org/ while FLN was sourced from the UCSF ZINC database (ZINC19360739) [54]. Molecular docking of FLN into β-CD cavity was carried out following the methods as reported previously [50]. The grid centre of docking coordinates were x = − 6.89 Å, y = − 7.65 Å and z = 4.34 Å. The grid dimensions were 54 Å , 56 Å and 44 Å in x, y and z-axes respectively.

## Results and discussion

### 2D ^1^H-^1^H ROESY spectrum of β-CD-FLN mixture and structure of the β-CD-FLN inclusion complex by NMR and molecular docking approaches

**Fig. 2:**
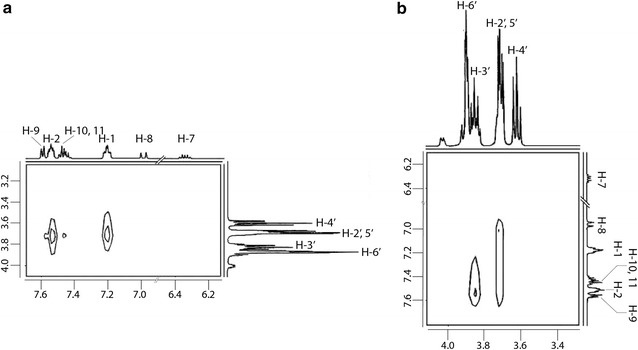
**a** and **b** Partial 2D ^1^H-^1^H ROESY (500 MHz) spectra of the mixture of β-CD and FLN showing interactions between FLN and β-CD cavity protons (*τ*_mix_ = 500 ms)

The understanding of host–guest supramolecular structure is important for the pharmaceutical industry for development of drug-CD based new formulations. In order to clearly establish the identity of the aromatic ring involved in complexation between β-CD and FLN, a 2D ^1^H-^1^H ROESY spectrum of the mixture of β-CD and FLN was analyzed. The NMR spectroscopic studies and assignments of β-CD and FLN protons are discussed in “^1^H NMR spectral assignments and chemical shift change data of β-CD” and “^1^H NMR spectral assignments and chemical shift change data of FLN” sections. Two-dimensional ^1^H-^1^H ROESY spectrum exhibited strong cross-correlation peak between the cavity protons of β-CD and the protons of the F-substituted aromatic ring of FLN thereby confirming the penetration of F-substituted aromatic rings into the β-CD cavity. The cross peaks between phenyl ring protons and β-CD cavity protons were also observed but these were relatively weak. It is apparent from the 2D ^1^H-^1^H ROESY spectrum that H-1 exhibited cross peak with only H-5′ while H-2 displayed cross peaks with both the H-3′ and H-5′ protons. The quality of 2D ^1^H-^1^H ROESY spectrum is not as good as required. Expansions of the parts of the 2D ^1^H-^1^H ROESY spectrum showing cross peaks between protons of aromatic rings of FLN and β-CD cavity protons are shown in Fig. 2. The full 2D ^1^H-^1^H ROESY spectrum displaying protons of β-CD and FLN and their NOE cross-correlation peaks close to proposed interaction site are presented in Additional file 1: Figure S1.

**Fig. 3:**
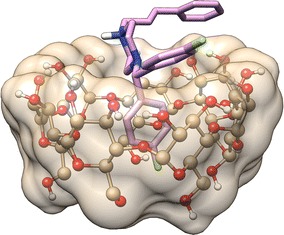
Computational best molecular docked conformation model of β-CD:FLN inclusion complex performed by Autodoc Vina 1.1.2 [53] showing penetration of F-substituted aromatic ring into β-CD cavity from wider rim side. The docking affinity was obtained to be − 5.4 kcal mol^−1^. Also, see Fig. 4 and Additional file 1: Figure S2 for other docked conformations obtained during docking. β-CD is shown as ball and stick with the surface while FLN is shown as stick bond. All atoms are shown in their elemental colour. Non-polar hydrogens are not shown for the sake of clarity. The figure was prepared using Chimera (http://www.cgl.ucsf.edu/chimera)

**Fig. 4:**
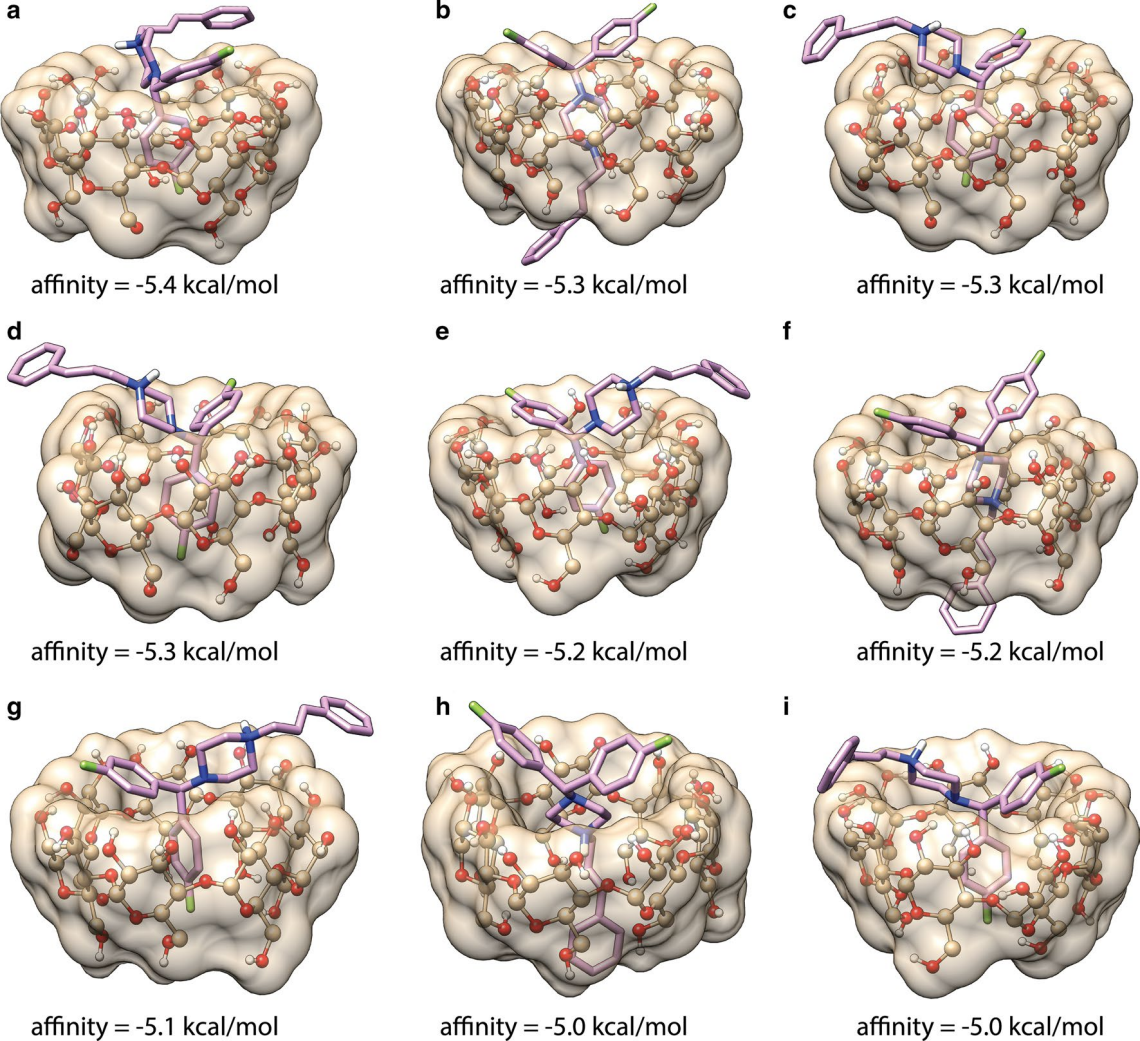
** a**–**i** Different binding conformations obtained during FLN docked into β-CD cavity. The docking affinity is shown under the model. β-CD is shown as ball and stick with the surface while FLN is shown as stick bond. All atoms are shown in their elemental colour. Non-polar hydrogens are not shown for the sake of clarity. The figure was prepared using Chimera (http://www.cgl.ucsf.edu/chimera)

On the basis of 1:1 stoichiometry of the β-CD-FLN inclusion complex (see “Stoichiometry and association constant of β-CD-FLN complex” section) and 2D ^1^H-^1^H ROESY spectral data, it can be inferred that F-containing aromatic ring preferentially enters into the β-CD cavity to form the inclusion complex. Also, the non-observance of the cross peak between H-3′ and H-1 (Fig. 2b) suggested the position of H-1 towards narrower rim side. The penetration from wider rim side would have brought H-1 closer to H-3′ also. It appears that there are interactions between the phenyl ring and β-CD but the amount is lower compared to complex formed involving F-containing aromatic ring. The penetration of FLN into β-CD cavity was reported to be from wider rim side based on 2D ^1^H-^1^H ROESY results [55] without clear inclusion architecture. The plausible mode of inclusion and structure of the β-CD-FLN inclusion complex cannot be achieved only from 2D ^1^H-^1^H ROESY spectral data and therefore, another approach was required. In order to understand the β-CD-FLN inclusion complex structure, computer-based molecular docking was performed using Autodock Vina 1.1.2 [53]. Molecular docking studies provide us not only the mode of inclusion but also the depth of penetration inside the β-CD cavity during complexation process. The best-docked model of β-CD:FLN complex is shown in Fig. 3. It is evident that the mode of penetration of FLN guest into the β-CD cavity was from the wider rim side and similar to 2D ^1^H-^1^H ROESY results the F-containing aromatic ring participates more favourably than phenyl ring. We compared our result with cetirizine dihydrochloride (CTZ) which has some structural similarity with FLN. CTZ, an antihistamine drug used to treat allergies, formed 1:1 inclusion complexes in which the penetration of CTZ into the β-CD cavity was from wider rim side [56]. Similarly our 2D ^1^H-^1^H ROESY and molecular docking approach together provide information about the penetration of FLN from the wider rim side of the β-CD cavity [56]. Moreover, F-containing aromatic ring of FLN positioned towards the narrower rim of the β-CD truncated cone, which is also observed in 2D ^1^H-^1^H ROESY spectrum containing the cross peak between H-1, H-2 and H-5′. In the other dockings conformations models, it is apparent that the phenyl ring also participates in complexation (Fig. 4). Interestingly, similar to 2D ^1^H-^1^H ROESY results, the phenyl ring protrudes on the opening of the narrower rim side of the β-CD cavity (Fig. 4b, f, h). The docking binding affinity for the best energy minimized β-CD:FLN complex was obtained to be − 5.4 kcal mol^−1^, which is favourable for such type of complex [50]. The ensemble of all possible computational docking conformations of β-CD:FLN complex is shown in Additional file 1: Figure S2. Based on molecular docking studies performed, it is apparent that all aromatic rings dock into the β-CD cavity but F-containing aromatic ring participates more favourably than the phenyl ring.

### ^1^H NMR spectral assignments and chemical shift change data of β-CD

The assignment of the β-CD protons, in the spectra of β-CD and FLN mixture, was made with the help of their ^1^H signals and 2D ^1^H-^1^H COSY spectral data [47]. Expansion of 2D ^1^H-^1^H COSY spectrum of an FLN:β-CD mixture showing β-CD regions are shown in Additional file 1: Figure S3. On the investigation of ^1^H NMR spectra of mixtures of β-CD and FLN, an upfield shift in H-3′ and H-5′ (located inside the central cavity) signals of β-CD was observed [47, 50–52, 56]. Other β-CD signals (H-1′, 2′, 4′, 6′) also exhibited shift changes but these were negligible compared to H-3′ and H-5′. In the presence of FLN, ∆δ for H-5′ were more pronounced than those of H-3′ signal of β-CD.

**Fig. 5:**
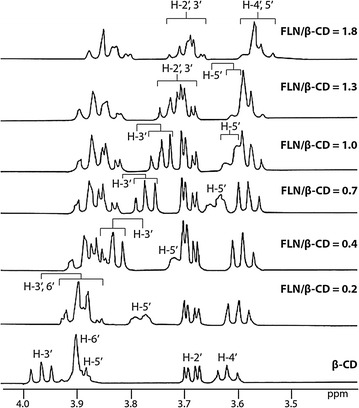
A part of ^1^H NMR spectra (500 MHz) showing protons of β-CD in the absence, as well as in the presence, of varying amount of FLN

The upfield shift of ^1^H signals located inside the cavity, namely H-3′ and H-5′, have been attributed to the magnetic anisotropy effect in the β-CD cavity due to the inclusion of groups rich in π-electrons [51]. The continuous upfield shift changes of ^1^H signals observed in H-3′ and H-5′ of β-CD in the ^1^H NMR spectra of β-CD-FLN mixtures thus confirm the formation of the inclusion complex between β-CD and FLN [47, 50–52, 56]. Expansions of part of ^1^H NMR spectra of pure β-CD and mixture of β-CD and FLN in varying amounts of FLN are displayed in Fig. 5 and their ∆δ data are listed in Table 1.

**Table 1:** ^1^H NMR (500 MHz) chemical shift change (Δδ) data for the β-CD protons in the presence of FLN

[FLN]/[β-CD]	H-1′	H-2′	H-3′	H-4′	H-5′	H-6′
0.2	− 0.056	− 0.001	− 0.105	− 0.022	− 0.117	− 0.009
0.4	− 0.067	− 0.003	− 0.157	− 0.033	− 0.184	− 0.019
0.7	− 0.078	− 0.005	− 0.203	− 0.043	− 0.239	− 0.045
1.0	− 0.084	− 0.008	− 0.227	− 0.058	− 0.275	− 0.052
1.3	− 0.086	− 0.011	− 0.244	− 0.060	− 0.294	− 0.053
1.8	− 0.088	− 0.007	− 0.258	− 0.062	− 0.319	− 0.057

Negative values indicate upfield shift changes

### ^1^H NMR spectral assignments and chemical shift change data of FLN

**Fig. 6:**
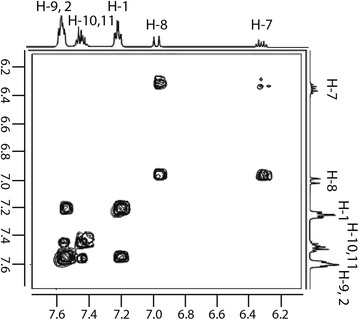
Part of the 2D ^1^H-^1^H COSY spectrum (500 MHz) of a mixture of β-CD and FLN, displaying through the bond interaction of aromatic protons of FLN

The resonance assignment of guest FLN aromatic ring protons in the free as well as host β-CD bound state were achieved using ^1^H NMR as well as 2D ^1^H-^1^H COSY spectral data. Part of the 2D ^1^H-^1^H COSY spectrum of the mixture of β-CD and FLN displaying through bond cross connection peaks between aromatic protons of FLN is shown in Fig. 6.

The aromatic protons were observed as three signals, a triplet at 7.20 ppm integrating for four protons, a multiplet at 7.45 ppm for three protons and a multiplet at 7.58 ppm for six protons. Fluorine has a slight ‘donor substituent’ effect in the benzene ring. For instance, ortho-, meta-, and para-proton signals of fluorobenzene appear at 6.99, 7.24 and 7.08 ppm, respectively. In styrene, ortho, meta and para protons are increasingly shielded. In order to elucidate the question on H-1 and H-2 assignment, we examined for intramolecular NOE cross peaks between H-2 and H-3 in ^1^H-^1^H ROESY spectrum. The triplet at 7.20 ppm (*J *= 8.6 Hz) was assigned to H-1 protons and it showed the ^1^H-^1^H COSY interaction with the multiplet at 7.58 ppm, which was ascribed to H-9 and H-2 protons. The observed shape of H-1 and H-2 (like triplets) are undoubtedly from ^1^H-^19^F cross coupling interactions. It is well known that, for fluorobenzene derivatives, the coupling constants 3*J*(H, F) = 6.2–10.1 Hz and 4*J*(H, F) = 6.2–8.3 Hz. The multiplet at 7.45 ppm was due to H-10, 11 protons. In FLN:β-CD mixtures, the signal for H-2 and H-9 separated and the nature of H-2 resembles a triplet. A doublet at 6.97 ppm (*J *= 16.0 Hz), which appeared in the aromatic region was ascribed to H-8, while the H-7 was found resonating as a merged doublet of the triplet at 6.32 ppm.

The aromatic protons of FLN were deshielded and pattern of their ^1^H NMR peaks splitting in presence of β-CD suggests some chiral differentiation of guest FLN by host β-CD [48, 49]. The ^1^H NMR signal for H-9, 2 which appeared as a merged signal in the spectrum of unbound FLN, separated in the spectra of some FLN:β-CD mixtures. The ^1^H NMR spectra of expanded aromatic regions of guest FLN in the bound as well as unbound with host β-CD is shown in Additional file 1: Figure S4. The ^1^H NMR shielding and deshielding pattern of β-CD and FLN protons in the bound state indicate the involvement of aromatic ring group in complexation [47] but the identity of the aromatic rings penetrating into β-CD cavity could not be achieved and therefore further studies were required. Two-dimensional ^1^H-^1^H ROESY and molecular docking studies further applied to understand β-CD-FLN inclusion complex structure (see “2D ^1^H-^1^H ROESY spectrum of β-CD-FLN mixture and structure of the β-CD-FLN inclusion complex by NMR and molecular docking approach” section).

### Stoichiometry and association constant of β-CD-FLN complex

**Fig. 7:**
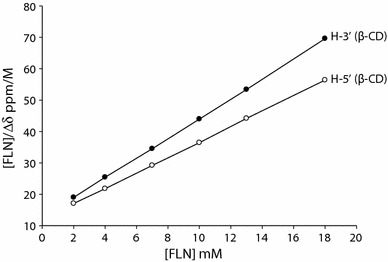
Scott’s plot showing 1:1 stoichiometry for the β-CD-FLN complex

Next, we wanted to determine the stoichiometry, association constant (*K*_a_) and the Gibb’s free energy (∆G) of the β-CD-FLN inclusion complex. The stoichiometry and *K*_a_ of the β-CD-FLN complex were established with the help of the Scott’s method [57]. In Scott’s equation,

1 $$ \left[ {\text{FLN}} \right]_{\text{t}} /\Delta \delta_{\text{obs}} = \, \left[ {\text{FLN}} \right]_{\text{t}} /\Delta \delta_{\text{c}} + { 1}/K_{\text{a}} \Delta \delta_{\text{c}} $$

where [FLN]_t_ is the molar concentration of the guest, Δδ_obs_ the observed chemical shift change for a given [FLN]_t_ concentration, Δδ_c_ the chemical shift change between a pure sample of complex and the free component at the saturation. The plot of Δδ for the β-CD protons (H-3′ and H-5′) against [FLN] in the form of [FLN]/Δδ_obs_ versus [FLN] appeared to be linear fits (Fig. 7) suggesting 1:1 stoichiometry for the β-CD-FLN inclusion complex. The slope of the plot (Fig. 7) is thus equal to 1/Δδ_c_ and the intercept with the vertical axis to 1/*K*_a_Δδ_c_ allowing the estimation of *K*_a_ to be 157 M^−1^, which is the average of two *K*_a_.

We were also interested to probe the differences between binding constants of two nearly similar structures. The binding constant of β-CD-CTZ complex was reported earlier to be 70 M^−1^ [56], which is nearly half of the binding constant calculated for β-CD-FLN complex. This could be due to the structural differences between CTZ and FLN. The ∆G associated during β-CD and FLN inclusion complex was calculated using standard Eq. (2):

2 $$ \Delta {\text{G }} = \, {-}{\text{ RTln}}K_{a} $$

where R is the universal gas constant (J mol^−1^ K^−1^), T is temperature (Kelvin) and *K*_*a*_ is the binding constant. The ∆G value was calculated to be − 12.65 kJ mol^−1^.

## Conclusions

The ^1^H NMR spectral data of pure FLN, pure β-CD and mixtures of β-CD and FLN in D_2_O confirmed the complexation between β-CD and FLN. The ^1^H NMR together with 2D ^1^H-^1^H COSY spectral data provided the resonance assignment of host and guest molecules. The stoichiometry, association constant and the Gibb’s free energy were determined using ^1^H NMR titration data. Two-dimensional ^1^H-^1^H ROESY spectral data together with computational molecular docking simulation studies confirmed that F-substituted aromatic ring of guest penetrates into the host β-CD cavity from the wider rim side. The tail end aromatic rings of guest FLN were proximal near to narrower rim side of truncated host β-CD cone. The splitting of the most of the aromatic ring protons of the FLN, in the presence of β-CD, suggests some chiral differentiation of guest FLN by host β-CD. The structural studies of FLN-β-CD inclusion complex may open new avenues for new drug formulation in the pharmaceutical industry.

## Additional file

**Additional file 1: Figure S1:** Full 2D ^1^H-^1^H ROESY spectrum (500 MHz) showing through space cross correlation peaks between β-CD protons and aromatic rings proton of FLN. **Figure S2**. Ensemble of different conformations obtained during FLN docked into β-CD cavity. The best docking conformation model shown as bond while other docking conformation modes are shown as wire frame. All atoms are shown in their elemental colour (β-CD and best docking conformer). The inclusion of guest FLN was from the wide rime side during all docking simulations performed. The phenyl ring of FLN are close to the narrow rim in some docking conformations. β-CD shown as ball and stick with surface while FLN shown as stick/wire bond. Non-polar hydrogens are not shown for sake of clarity. The figure was prepared using Chimera (http://www.cgl.ucsf.edu/chimera). **Figure S3**. Expanded region of 2D ^1^H-^1^H COSY spectrum (500 MHz) of FLN:β-CD mixture showing β-CD region. The assignments of β-CD protons namely H-1′, H-2′, H-3′, H-4′, H-5′ and H-6′ was made with the help of ^1^H NMR and 2D ^1^H-^1^H COSY cross correlation peaks. **Figure S4**. Expansion of part of ^1^H NMR spectra (500 MHz) of FLN protons in the presence, as well as in the absence, of β-CD.
