# Supplementary material for: Molecular recognition of flunarizine dihydrochloride and β-cyclodextrin inclusion complex by NMR and computational approaches
Source: Chem Cent J. 2018 Mar 28;12:33. doi: 10.1186/s13065-018-0395-4 (PMC5871610; doi:10.1186/s13065-018-0395-4)
Supplement: Supplementary file 1 — Additional file 1: Figure S1: Full 2D 1H-1H ROESY spectrum (500 MHz) showing through space cross correlation peaks between β-CD protons and aromatic rings proton of FLN. Figure S2. Ensemble of different conformations obtained during FLN docked into β-CD cavity. The best docking conformation model shown as bond while other docking conformation modes are shown as wire frame. All atoms are shown in their elemental colour (β-CD and best docking conformer). The inclusion of guest FLN was from the wide rime side during all docking simulations performed. The phenyl ring of FLN are close to the narrow rim in some docking conformations. β-CD shown as ball and stick with surface while FLN shown as stick/wire bond. Non-polar hydrogens are not shown for sake of clarity. The figure was prepared using Chimera (http://www.cgl.ucsf.edu/chimera). Figure S3. Expanded region of 2D 1H-1H COSY spectrum (500 MHz) of FLN:β-CD mixture showing β-CD region. The assignments of β-CD protons namely H-1′, H-2′, H-3′, H-4′, H-5′ and H-6′ was made with the help of 1H NMR and 2D 1H-1H COSY cross correlation peaks. Figure S4. Expansion of part of 1H NMR spectra (500 MHz) of FLN protons in the presence, as well as in the absence, of β-CD. [file 13065_2018_395_MOESM1_ESM.docx]

**Additional Information**

**Molecular recognition of flunarizine dihydrochloride and β-cyclodextrin inclusion complex by NMR and computational approach**

Santosh Kumar Upadhyay^1^ and Syed Mashhood Ali^2^

^1^CSIR-Institute of Genomics & Integrative Biology, New Delhi-110020, India.

^2^Department of Chemistry, Aligarh Muslim University, Aligarh-202 002, UP, India.


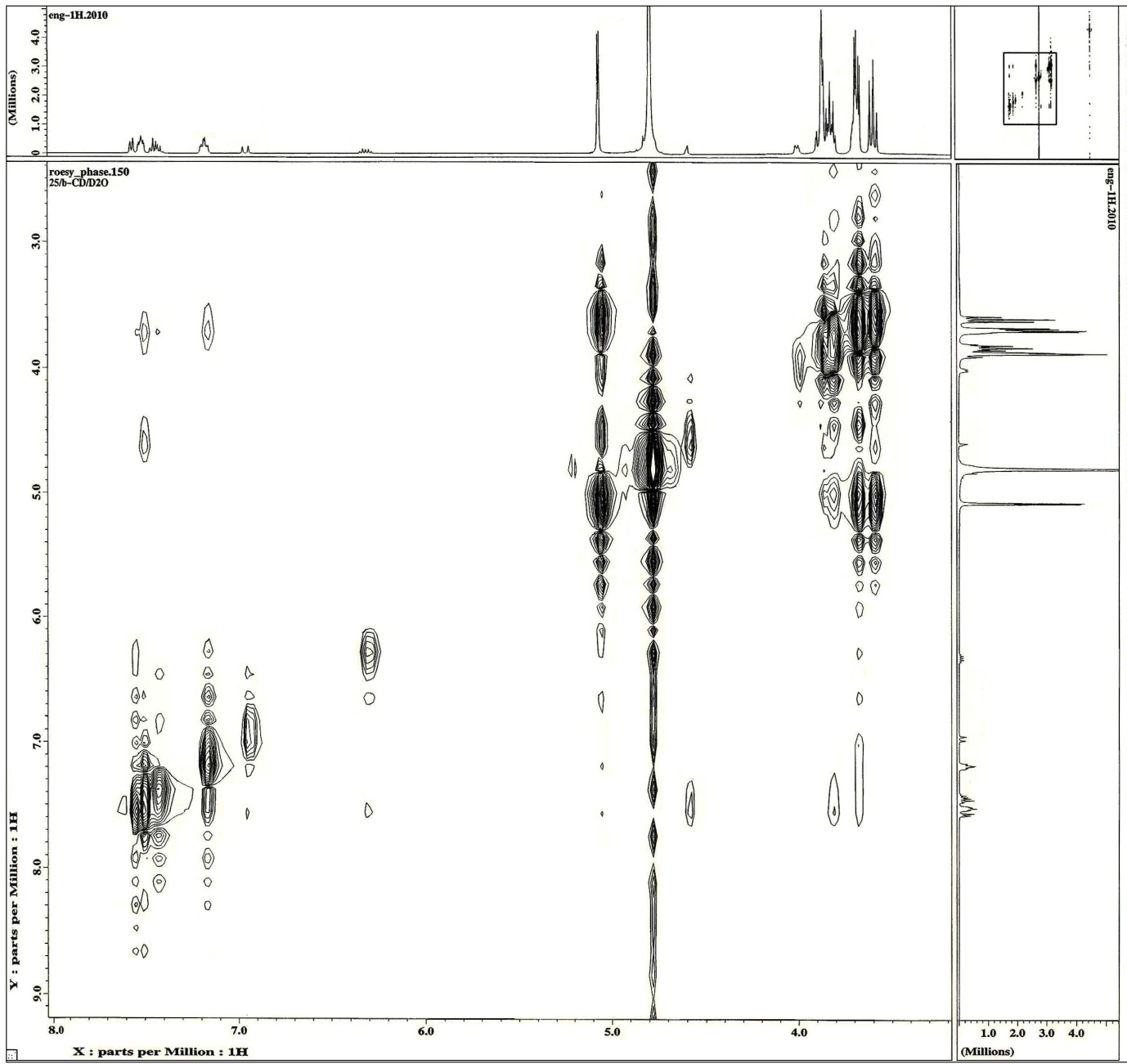


**Figure S1.** Full 2D ^1^H-^1^H ROESY spectrum (500 MHz) showing through space cross correlation peaks between β-CD protons and aromatic rings proton of FLN.


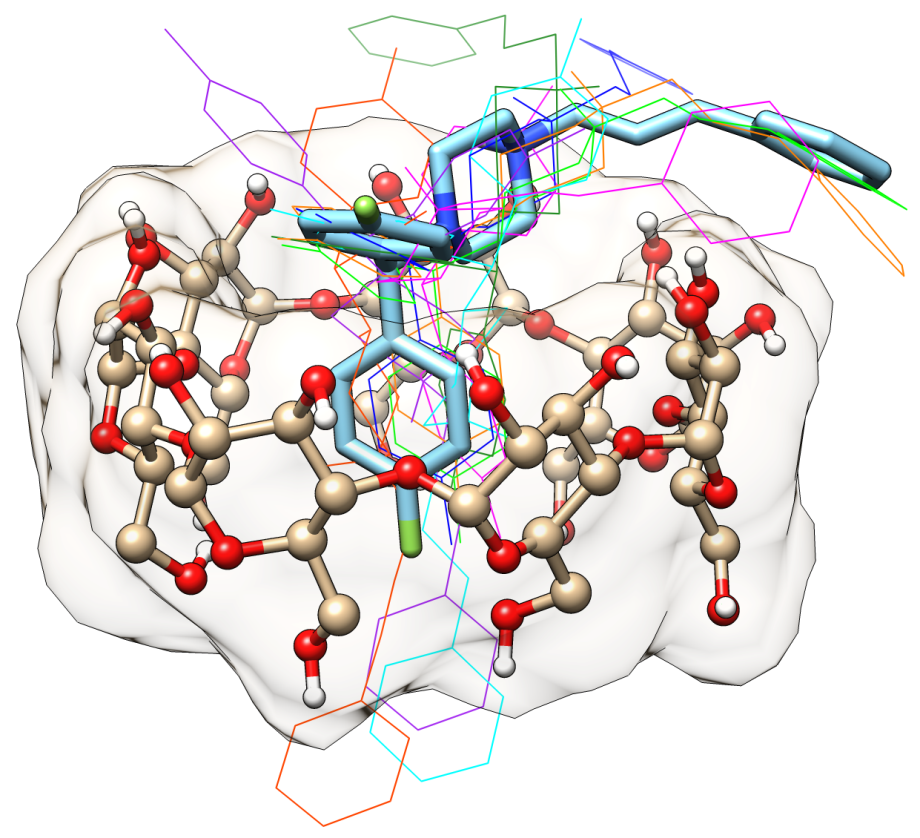


**Figure S2:** Ensemble of different conformations obtained during FLN docked into β-CD cavity. The best docking conformation model shown as bond while other docking conformation modes are shown as wire frame. All atoms are shown in their elemental colour (β-CD and best docking conformer). The inclusion of guest FLN was from wider rime side during all docking simulation performed. The phenyl ring of FLN are close to narrower ring in some docking conformations. β-CD shown as ball and stick with surface while FLN shown as stick/wire bond. Non-polar hydrogens are not shown for sake of clarity. The figure was prepared using Chimera (<http://www.cgl.ucsf.edu/chimera)>.


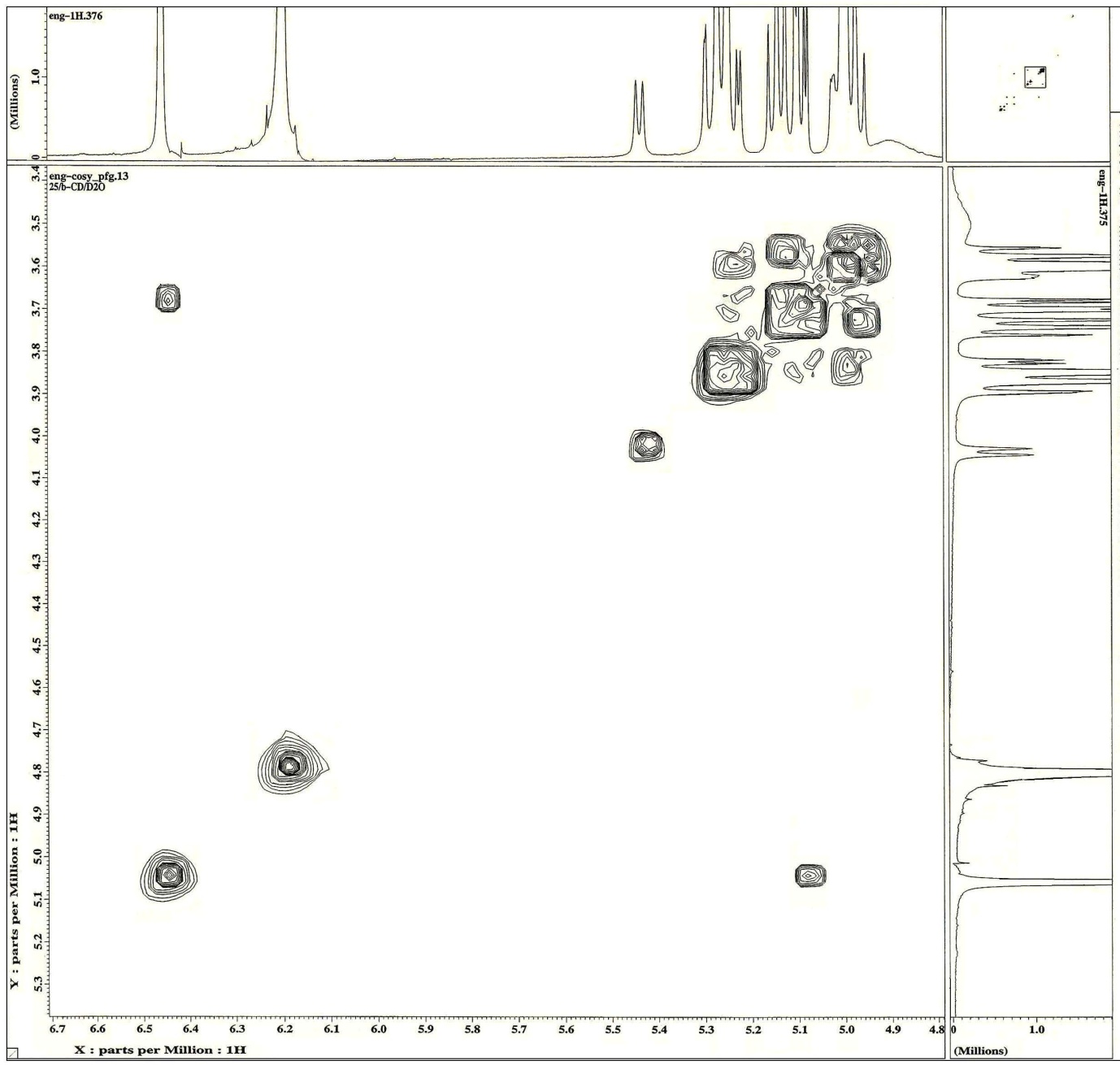


**Figure S3.** Expended region of 2D ^1^H-^1^H COSY spectrum (500 MHz) of FLN:β-CD mixture showing β-CD region. The assignments of β-CD protons namely H-1’, H-2’, H-3’, H-4’, H-5’ and H-6’ was made with the help of 2D ^1^H-^1^H COSY cross correlation peaks.


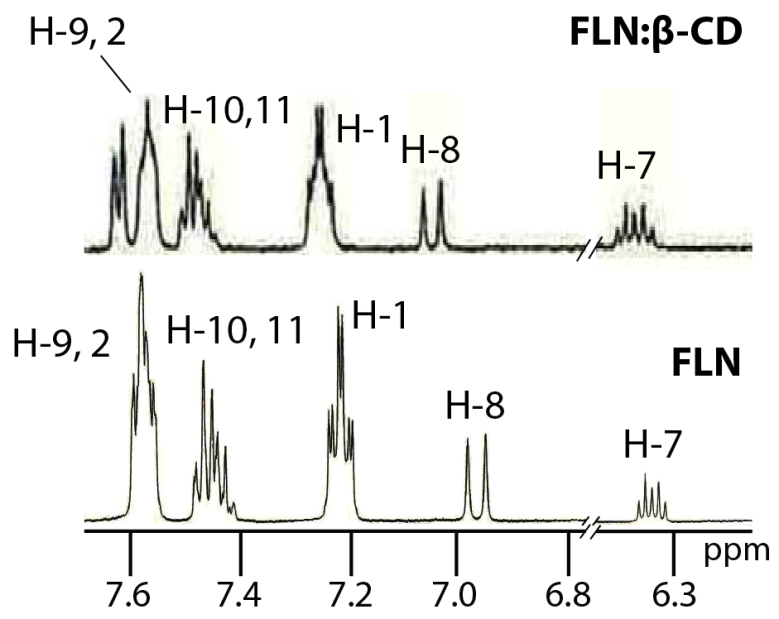


**Figure S4.** Expansion of parts of ^1^H NMR spectra (500 MHz) of FLN protons in the presence, as well as in the absence, of β-CD.
